# Renal Tubular Acidosis and Hypokalemic Paralysis as a First Presentation of Primary Sjögren's Syndrome

**DOI:** 10.1155/2018/9847826

**Published:** 2018-10-16

**Authors:** Arun Sedhain, Kiran Acharya, Alok Sharma, Amir Khan, Shital Adhikari

**Affiliations:** ^1^Nephrology Unit, Department of Medicine, Chitwan Medical College, Bharatpur, Chitwan, Nepal; ^2^Department of Medicine, Chitwan Medical College, Bharatpur, Chitwan, Nepal; ^3^Department of Renal Pathology & Electron Microscopy, National Reference Laboratory, Dr. Lal Pathlabs Ltd, New Delhi, India; ^4^Pulmonology and Critical Care Unit, Department of Medicine, Chitwan Medical College, Bharatpur, Chitwan, Nepal

## Abstract

Sjögren's syndrome is an autoimmune disease with multisystem involvement and varying clinical presentation. We report the clinical course and outcome of a case who presented with repeated episodes of hypokalemia mimicking hypokalemic periodic paralysis and metabolic acidosis, which was later diagnosed as distal renal tubular acidosis secondary to primary Sjögren's syndrome. A 50-year-old lady, who was previously diagnosed as hypokalemic periodic paralysis, presented with generalized weakness and fatigue. She was found to have severe hypokalemia with normal anion-gap metabolic acidosis consistent with distal renal tubular acidosis. Subsequent evaluation revealed Sjögren's syndrome as the cause of her problems. Kidney biopsy done to evaluate significant proteinuria revealed nonproliferative morphology with patchy acute tubular injury and significant chronic interstitial nephritis. The patient responded well to potassium supplementation and oral prednisolone. Presentation of this case highlights the necessity of close vigilance while managing a case of repeated hypokalemia, which could be one of the rare clinical manifestations of Sjögren's syndrome.

## 1. Introduction

Sjögren's syndrome (SS) is a slowly progressing autoimmune disease characterized by lymphocytic infiltration of the exocrine glands, mainly the lacrimal and salivary glands, resulting in impaired secretory function. The disease has an estimated prevalence of 0.3 to 1 per 1000 persons and a peak incidence at approximately 50 years of age with female-to-male predominance of 9:1 [1].

Renal involvement is seen in 5% of patients with SS, with the most common of which being chronic interstitial nephritis [2–4]. Renal tubular acidosis (RTA) occurs in up to 25% of patients with the disease [5], most of which are usually asymptomatic. We report a case requiring multiple hospital admissions with a clinical diagnosis of hypokalemic periodic paralysis previously presented to us with severe hypokalemia associated with metabolic acidosis, which was later diagnosed to be secondary to Sjögren's syndrome.

## 2. Case Report

A 50-year-old woman presented to the Emergency Department (ED) of Chitwan Medical College, Bharatpur, Chitwan, Nepal, with the history of weakness of both lower limbs for two days that was preceded by muscle cramps of three days' duration. Her weakness was insidious in onset and gradually progressive in nature affecting the upper limbs by next day with no history of altered sensorium, seizure, and bladder or bowel involvement. Her past medical history was positive for repeated hospital admissions following episodes of weakness and fatigue associated with hypokalemia for the past three years, which was managed in the line of hypokalemic periodic paralysis that responded well to supplemental potassium alone. She also had similar problems episodically for the past three years requiring repeated hospital admissions. The lady also had a history of drooping of her bilateral eyelids, foreign body sensation in the eyes, dry mouth, and recurrent muscular weakness for the past three years. She denied history of vomiting and intake of diuretics, alcohol, or laxatives. Previous medical records revealed negative results for antibody against acetylcholine receptor that ruled out myasthenia gravis.

On physical examination, vital signs were within normal limit and higher mental functions were intact. Her oral cavity was dry and there was no lymphadenopathy. Motor power was 3/5 on the lower limbs and 4/5 on the upper limb affecting both proximal and distal group of muscles. Deep tendon reflexes were diminished bilaterally. There was no sensory deficit and cranial nerve examination was unremarkable. Cardiovascular, respiratory, gastrointestinal, and thyroid examination findings were normal.

She was found to have hypokalemia (documented serum K+ of 1.6 meq/L; normal range 3.5-5.5 meq/L) (Table 1). ECG showed a sinus bradycardia with global T wave inversion and the presence of subtle U wave.

In the Emergency Department, the patient was started on intravenous potassium supplementation at the rate of 20 meq/hour via central line and was admitted to the intensive care unit (ICU), where treatment was continued and serial monitoring of potassium level was done. Consecutive serum potassium levels at 6^th^, 12^th^, and 48^th^ hour after initiation of treatment were 1.75 mmol/L, 2.1 mmol/L, and 3.7 mmol/L, respectively. Intravenous magnesium supplementation and injection sodium bicarbonate were also given. After 12 hours of treatment, her clinical condition improved significantly with normalization of the muscle power.

With the urinary pH of 5.0, negative urine culture, no history of diuretic usage, vomiting, and diarrhea, and the arterial blood gas (ABG) showing hyperchloremic normal anion-gap metabolic acidosis in a patient with severe hypokalemia (serum potassium 1.7 mmol/L), the diagnosis of distal renal tubular acidosis (DRTA) was made. With the history of xerostomia and xerophthalmia without any secondary causes for them, SS was suspected, which was later confirmed by the significantly raised titers of anti-Ro/SSA and/or anti-La/SSB antibodies and positive Schirmer test (4.8 mm in 5 minutes) as per the latest classification criteria [6].

She was started on oral prednisolone at 1 mg/kg/day after which ptosis showed partial recovery in the first 7 days. She was discharged with the same dose of prednisolone and was advised for regular follow-up in nephrology clinic.

**Figure 1:**
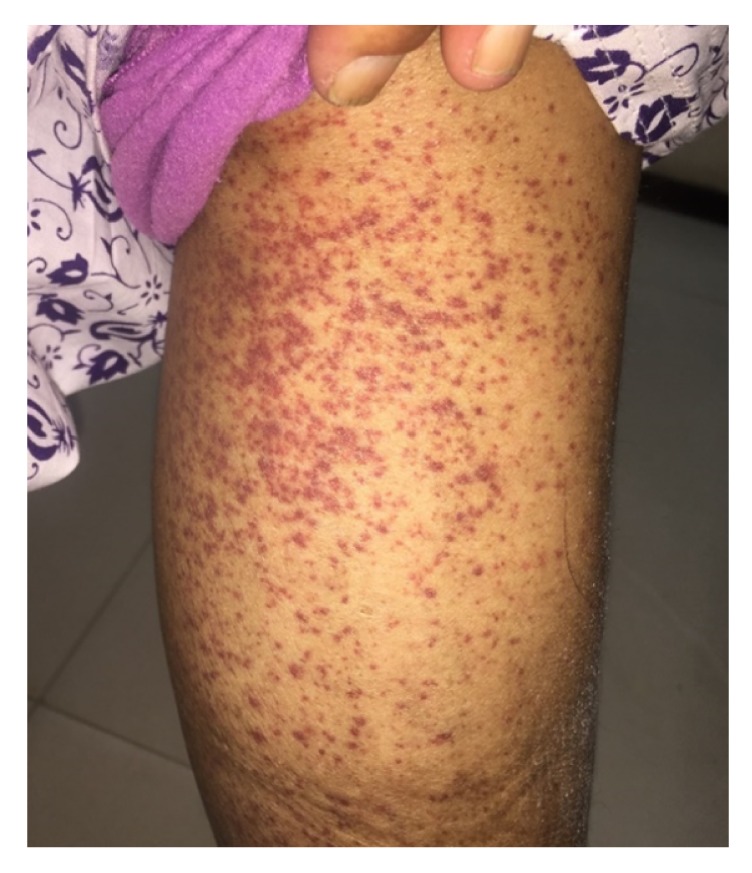
Palpable purpura on the lower limb.

**Figure 2:**
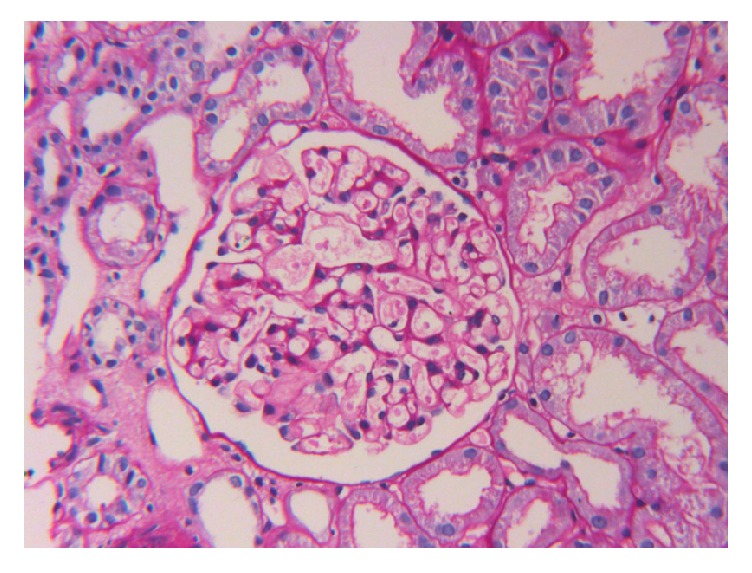
Photomicrograph from renal biopsy showing an unremarkable appearing glomerulus (PAS X 200).

**Figure 3:**
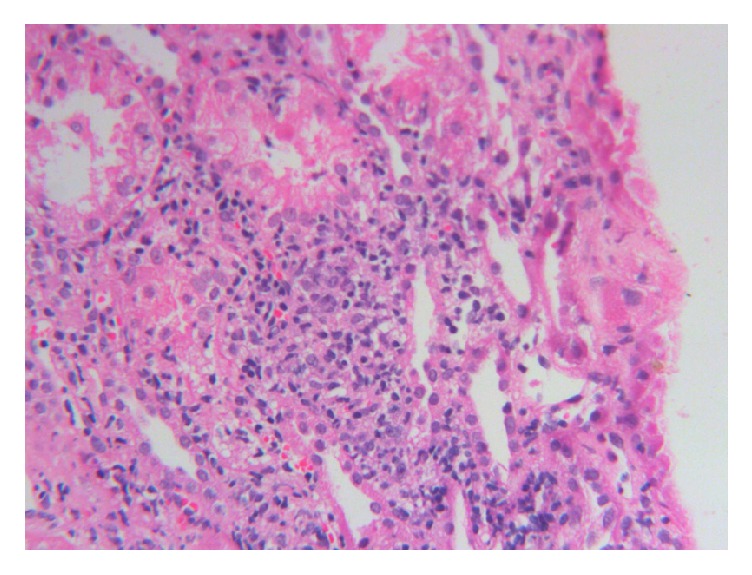
Photomicrograph showing dense chronic lymphoplasmacytic interstitial inflammation (H&E X 160).

**Figure 4:**
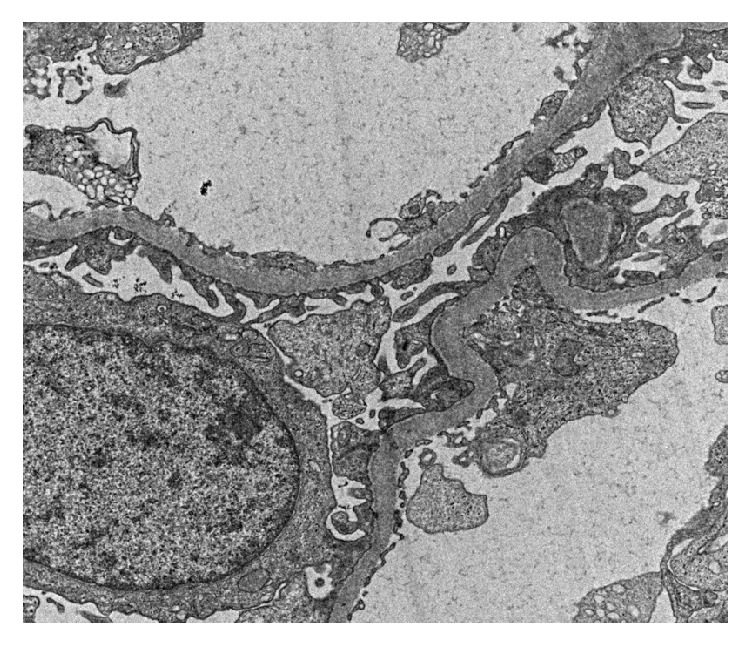
Electron micrograph showing glomerular capillaries with well-preserved foot processes of visceral epithelial cells (uranyl acetate and lead citrate X 3000).

The patient attended the nephrology clinic after 7 days with palpable purpuric rashes in both of the lower limbs associated with minimal pedal edema (Figure 1). She was reevaluated and skin biopsy was suggested, but she refused it. She was found to have normal hemogram and bleeding profile and negative perinuclear antineutrophil cytoplasmic antibodies (P-ANCA), antineutrophil cytoplasmic antibodies (C-ANCA), and cryoglobulins. Urine examination showed 2+ albumin without associated hematuria and 24-hour urinary protein was 1600 mg, for which she underwent kidney biopsy. Light microscopy showed nonproliferative glomerular morphology (Figure 2) with patchy acute tubular injury and multifocal chronic interstitial inflammation (Figure 3). Direct immunofluorescent examination revealed no significant glomerular immune deposits. Transmission electron microscopy revealed relatively well-preserved visceral epithelial cell foot processes (Figure 4) and no evidence of glomerular or extraglomerular electron dense deposits. Endothelial tubuloreticular inclusions were not seen. Proximal tubular epithelial cells did not reveal abnormal inclusions or giant mitochondria.

The patient is on regular follow-up for the last eight months and the oral steroids is getting tapered gradually. She is doing well with improvement in proteinuria, resolution of acidosis, and hypokalemic episodes.

## 3. Discussion

Our patient presented with the complaints of muscle weakness secondary to severe hypokalemia (serum K+ 1.6 meq/L). On further evaluation in our center, she had normal anion-gap hyperchloremic metabolic acidosis (HCMA). Despite lack of a more comprehensive evaluation, the biochemical findings of renal potassium loss in association with HCMA were supportive of the diagnosis of distal renal tubular acidosis (RTA) in our patient. Further history obtained from the patient revealed that she had a history of foreign body sensation in the eyes and dry mouth for the past three years, which prompted us to evaluate for the possibility of Sjögren's syndrome as the root cause of her recurrent clinical problems. Significantly raised titers of anti-Ro/SSA and anti-La/SSB antibodies and positive Schirmer test confirmed Sjögren's syndrome. She later developed significant proteinuria, for which kidney biopsy was done showing nonproliferative morphology with patchy acute tubular injury and focal chronic interstitial inflammation. She was started with oral prednisolone and was kept on regular follow-ups with significant clinical improvements.

Sjögren's syndrome is a systemic autoimmune disorder characterized by a unique set of signs and symptoms predominantly caused by a cell-mediated autoimmunity against exocrine glands [7]. Systemic manifestations occur in approximately 30 to 40% of the patients with primary Sjögren's syndrome [2]. Lymphocytic infiltration can cause interstitial nephritis, autoimmune primary biliary cholangitis, and obstructive bronchiolitis. Immune complex deposition can result in palpable purpura, cryoglobulinemia-associated glomerulonephritis, interstitial pneumonitis, and peripheral neuropathy [8].

The most common affected nonexocrine organ in Sjögren's syndrome is kidney with the prevalence ranging between 2 and 67% [9, 10]. Most common form of renal involvement in Sjögren's syndrome is interstitial nephritis followed by distal renal tubular acidosis (dRTA), nephrogenic diabetes insipidus, and different forms of glomerular diseases, of which membranoproliferative glomerulonephritis (MPGN) and membranous nephropathy (MN) are the most common [3, 4]. Although dRTA is common in Sjögren's syndrome, it is usually asymptomatic and in most cases it remains undetected. Hypokalemia is the most common electrolyte abnormality in patients with dRTA. The causes of hypokalemia include decreased distal tubular Na+ delivery, secondary hyperaldosteronism, defective H+-K+ ATPase, and bicarbonaturia. Hypokalemic paralysis seen in SS is rare and may sometimes mimic hypokalemic periodic paralysis (HPP). However, there are case reports of single presentation of severe hypokalemic paralysis, which was later confirmed as Sjögren's syndrome [11].

A diagnosis of primary Sjögren's syndrome is often considered based on the classic symptoms of mouth and eye dryness, fatigue, and pain [2]. However, systemic complications sometimes provide the first clue to the disease as seen in our case, in which the presenting complaint was muscle weakness secondary to severe hypokalemia and metabolic acidosis. Anti-SSA antibodies (antibodies against Sjögren's syndrome–related antigen A) are present in two-thirds of patients and should be assessed in all suspected cases of primary Sjögren's syndrome. Biopsy of minor salivary glands is typically recommended for establishing a diagnosis of primary Sjögren's syndrome in the absence of anti-SSA antibodies. Schirmer's test to assess the ocular dryness is a useful examination. A recent set of classification criteria for SS were published by the ACR/EULAR in 2016 [6] and the score of ≥4 is required for the diagnosis.

Management of primary SS is symptomatic. In the acute setting, when the patient presents with hypokalemia, the priority will be to reverse the severe hypokalemia with intravenous potassium supplementation, followed by correction of the underlying acidosis. Long-term use of potassium supplementation might be required for majority of the patients. Use of muscarinic agonists (pilocarpine hydrochloride and cevimeline hydrochloride) is recommended for the treatment of oral dryness and, to a lesser extent, ocular dryness [12]. Neuropathic pain in patients with primary Sjögren's syndrome is typically treated with gabapentin, pregabalin, or duloxetine. Although no immunomodulatory drug has proved to be efficacious in primary SS, combination of corticosteroids and other immunosuppressive drugs has been reported to slow the progression of renal damage in Sjögren's syndrome [13]. Agents that are commonly used include hydroxychloroquine, prednisone, methotrexate, mycophenolate sodium, azathioprine, and cyclosporine. Few biologic agents have been rigorously studied in primary SS, and none have shown significant efficacy in multiple studies [14, 15]. The heterogeneity in the etiopathogenesis and clinical manifestation of the disease, in conjunction with a variable response to clinical therapeutics, warrants a more individualized approach to achieve improved long-term outcomes in patients with primary SS.

Our patient had repeated episodes of hypokalemia and metabolic acidosis in the past, which responded symptomatically to potassium supplementation alone. Thus, she was labelled as a case of hypokalemic periodic paralysis but detailed workup for the etiopathogenesis of her problem was missed.

## 4. Conclusion

Although Sjögren's syndrome might have a varying clinical presentation, presentation of a person with renal symptoms in the form of hypokalemia as the first symptom might create the confusion to reach the diagnosis. This case highlights the importance of high index of suspicion for possibility of Sjögren's syndrome, especially in the middle-aged females, who present with hypokalemia and metabolic acidosis.

## Figures and Tables

**Table 1:** Laboratory and biochemical parameters at presentation.

**Test**	**Result**
Hb	10.0 (g/dl)

WBC	5600 (per mm^3)^

Platelets	298,000 (per mm3)

ESR	67 (mm/1^st^ hour)

Serum Na^+^	148 (mEq/L)

Serum K^+^	1.6 (mEq/L)

Serum Urea	29 (mg/dL)

Serum Creatinine	1.0 (mg/dL)

Random blood sugar	130 (mg/dL)

Serum Magnesium	2.5 (mg/dL)

Serum Calcium	8.36 (mg/dL)

Serum pH	7.20

pCO_2_	18.8 (mmHg)

HCO_3_	7.1 (mEq/L)

pO_2_	89 (mmHg)

Serum Chloride	130 (mmol/L)

Anion Gap	11.9 (mmol/L)

Serum Vitamin 25(OH) D	6.40 (ng/ml)

Parathyroid hormone	145 (pg/ml)

TSH	8.74 (mIU/ml)

Urine pH	5.0

Urine K^+^	34.6

HIV, HBsAg, Anti-HCV	Negative

Hb: hemoglobin, ESR: erythrocyte sedimentation rate, RBS: random blood sugar, TSH: thyroid stimulating hormone. Serum anion gap = Na – (Cl + HCO3).
